# The relationship between depression and adjustment disorder among a clinical sample of college freshmen: the mediating roles of resilience and perceived social support

**DOI:** 10.3389/fpsyt.2026.1833604

**Published:** 2026-06-05

**Authors:** Shuai Zhang, Xiaoli Fan, Xinyue Shao, Zhao Dong, Yunyun Qiao, Xueli Guo, Hongwei Zhang, Hua Guo, Lingzi Fan

**Affiliations:** Pediatric Psychology Department, Zhumadian Second People’s Hospital, Zhumadian, China

**Keywords:** adjustment disorder, college freshmen, depression, mediating role, perceived social support, resilience

## Abstract

**Background:**

This study aimed to examine the association between depression and adjustment disorder in a clinical sample of college freshmen, and to investigate the mediating roles of resilience and perceived social support in this relationship.

**Methods:**

A cross-sectional survey was conducted among 402 college freshmen enrolled in the Pediatric Psychology Department of Zhumadian Second People’s Hospital. General data, the Patient Health Questionnaire (PHQ-9), Connor-Davidson Resilience Scale (CD-RISC), Perceived Social Support Scale (PSSS) and Adjustment Disorder–New Module 20 (ADNM-20) were completed by the participants. Descriptive, chi-square, and Pearson correlation analyses were conducted to characterize the sample and assess bivariate relationships, as appropriate for variable type. Hierarchical linear regression and path analysis were employed to examine the mediating roles of resilience and perceived social support between depression and adjustment disorder.

**Results:**

① Chi-square revealed no significant differences in adjustment disorder based on gender, age, only child status, childhood trauma experience, and educational level (P > 0.05). ② Pearson correlation analysis demonstrated significant relationships among the main study variables. Depression showed a strong positive correlation with adjustment disorder (r=0. 574, p<0.001), while both resilience (r=-0.653, p<0.001) and perceived social support (r=-0.550, p<0.001) were negatively correlated with adjustment disorder. ③ Hierarchical linear regression analysis indicated that the level of depression positively predicted adjustment disorder (β=0.278, P<0.001), whereas resilience (β=-0.459, P<0.001) and perceived social support (β=-0.364, P<0.001)negatively predicted adjustment disorder among college freshmen.

**Conclusions:**

In a clinical sample of college freshmen, the impact of depression on adjustment disorder is partially mediated by resilience and perceived social support. Our results supported a model in which depression contributed to adjustment disorder both directly and by eroding personal resilience and perceptions of social support.

## Introduction

Adjustment disorder refers to an adverse emotional or behavioral response to one or more identifiable stressors (1). A diagnosis of adjustment disorder is characterized by the onset of emotional and behavioral symptoms due to a stressful event within three months of the occurrence of a stressor; the symptoms cause significant distress exceeding what is expected from the stressor, or result in significant impairment in social, interpersonal, occupational, educational, or other functional areas. Students face many challenges when transitioning to a university environment, such as living independently away from home, health and time management, and adjusting study habits to fit the university environment (2). These challenges make college freshmen prone to adjustment problems, resulting in students exhibiting negative emotional symptoms such as depression, anxiety, and stress, which further affect their mental health (3). The prevalence of adjustment problems in college freshmen is higher than that in the general population and occurs more frequently at the beginning of the semester (4, 5). Multiple factors, such as academic workload, interpersonal challenges, and intense competition, are associated with students’ increased stress and adjustment problems; long-term stress may lead to serious consequences, especially adjustment disorders (6). Thus, adjustment disorder is a problem that cannot be ignored in the context of college students’ mental health.

Previous studies have found that depression is closely related to adjustment disorder (7, 8). Adjustment disorder and major depressive disorder share similar symptom profiles and differ primarily in symptom severity and prognosis (9). Genetic studies have also revealed a shared genetic etiology between adjustment disorder and major depressive disorder (10). Depression and adjustment problems are important factors affecting the physical and mental health of college students. As a negative emotion, depression can affect the physical and mental health of college students, mainly manifested as significant and sustained depression and loss of interest (11). The causes of adolescent depression are complex, with low recognition rates, low cure rates, and high suicide rates, which seriously endangers the physical and mental health and safety of adolescents. Depression in college students not only affects individual social functioning, academic performance, and interpersonal communication, but also has a greater impact on society (12). Despite the clinical importance of distinguishing between these two conditions, little is known about how depressive symptoms functionally influence the development and severity of adjustment disorder, particularly through potential mediating mechanisms.

Domestic and foreign studies have confirmed that some protective factors and successful stress management strategies are effective in alleviating stress and buffering negative stress responses (8, 13). Resilience refers to an individual’s ability to recover from a negative or painful event after suffering adversity or trauma, and adapt to the environment quickly by actively changing their mentality. Perceived social support refers to the emotional experience and satisfaction generated by an individual being respected, supported, and understood in society, which is closely related to an individual’s subjective feelings. The positive effects of resilience and social support on adaptability have been well-documented (14). Social support, particularly perceived emotional support, has been shown to counteract the harmful effects of stressful life events on the risk of depression and depressive symptoms (15). A cross-sectional study of military students (16) found that resilience was closely related to higher levels of adaptability, with social support most closely related to resilience. Self-Determination Theory and the associated Basic Psychological Needs Theory emphasize that a supportive environment is essential for healthy development and well-being by fulfilling innate psychological needs. It provides a theoretical basis for hypothesizing that depression might affect adjustment by undermining the perception of such a supportive environment (i.e., perceived social support) and the internal capacity for self-regulation (i.e., resilience), thereby leading to manifest adjustment difficulties.

Several studies have documented the complex interplay between depression, resilience, perceived social support, and adjustment disorders (17–19). Specifically, individuals with high levels of perceived social support tend to exhibit greater resilience, which in turn buffers the adverse impacts of negative events, mitigates associated negative emotions, alleviates depressive symptoms, and reduces the risk of mental health disorders (17, 20). Furthermore, individuals with high resilience can better adapt to the external environment, make fuller use of personal resources (such as social support), achieve good development, and improve life satisfaction (18). Additionally, increased social support has been shown to promote the development of mental resilience (19). Collectively, these findings highlight the intricate relationships among depression, resilience, perceived social support, and adjustment disorders.

However, prior research has predominantly focused on adjustment disorders and their influencing factors in individuals with depression, with few studies investigating the underlying mediating mechanisms. Notably, resilience and perceived social support—key factors affecting adjustment disorders in adolescents—have received limited attention regarding their specific pathway mechanisms. Exploring the serial mediating roles of resilience and perceived social support in the relationship between depression and adjustment disorders may offer novel insights for preventing maladaptive behaviors and promoting mental health among adolescents.

This study employed path analysis to examine the relationships between depression, resilience, perceived social support, and adjustment disorder among college freshmen. It aimed to elucidate the mediating roles of resilience and perceived social support in the relationship between depression and adjustment disorder, thereby identifying potential psychosocial mechanisms for informing targeted interventions.

## Materials and methods

### Participants

Zhumadian Second People’s Hospital (Zhengzhou University Affiliated Brain Disease Hospital), a major mental health institution in southern Henan, served as the recruitment site. Participants were randomly selected from among college freshmen who sought help at this hospital for adjustment problems. The study was conducted through the Department of Pediatric Psychology from May 2023 to December 2023.

#### Inclusion criteria

(1) college freshmen enrolled no more than three months prior to the study; (2) aged ≥16 years old; and (3) volunteered to participate in the study.

#### Exclusion criteria

(1) diagnosed with other organic diseases and mental disorders, (2) patients with cognitive dysfunction and intellectual disability, and (3) refusal to cooperate with the investigator. For participants under 18 years, written informed consent was obtained from parents or legal guardians, along with written assent from the participants themselves. Adult participants provided their own written informed consent. Ethical approval for this study was granted by the Ethics Committee of Zhumadian Second People’s Hospital (Approval No: Keshen-2023-005-01) in May 2023.

A simple random sampling method was employed to recruit participants for this study. The sample size was determined using the formula for cross-sectional studies: N = Z² * [P(1-P)]/E², where Z = 1.96 (corresponding to a 95% confidence level), P = 0.5 (maximum expected proportion), and E = 0.05 (margin of error). This calculation yielded a minimum required sample size of 385 participants. To account for potential invalid responses, we increased this target to 402 participants.

After quality control procedures, 390 valid questionnaires were retained from the 402 distributed, resulting in a valid response rate of 97.01%. The final sample of 390 participants demonstrated the following characteristics: age range 16–21 years (mean 18.11 ± 0.61); 69.23% male (n=270), 30.77% female (n=120); 44.62% only children (n=174), 55.38% not only children (n=216); 53.33% city residents (n=208), 46.67% country residents (n=182); 4.36% reporting childhood trauma experiences (n=17), 95.64% reporting no such experiences (n=373). Regarding primary childhood caregivers, 82.05% were raised by both parents (n = 320), 7.69% by the father or mother alone (n = 30), and 10.26% by grandparents or maternal grandparents (n = 40). The annual household income for 51.79% (n = 202) was below 100, 000 yuan and for 48.21% (n = 188) above 100, 000 yuan. Educational level was professional high school for 5.64% (n =22) and bachelor or college for 94.36% (n =368).

### Materials

#### General information questionnaire

A self-prepared general information questionnaire was administered, including gender, age, educational level, only child or not, growing up environment, family economic situation, childhood (before the age of 16) primary caregivers, and childhood (before the age of 16) traumatic experiences (domestic violence, assault, abuse, abandonment, and neglect).

#### Adjustment disorder--new module 20

This scale was developed by Glaesmer et al. (21) to assess the presence and severity of adjustment disorder. It contains 20 items and six factors, of which over-attention and maladaptation are the core factors, and four accessory symptom factors, including anxiety, depression, impulsivity, and avoidance. Item 20 is the functional assessment item. A Likert 4-level scoring system was adopted, with the score calculated by dividing the total score for each symptom by the number of items for the corresponding symptom. Symptom severity was assessed by the total score for all items, with a recommended clinical cutoff score of 47.5 (21). The internal consistency (Cronbach’s α) for the ADNM-20 in the present sample was 0.92.

#### Connor-Davidson resilience scale

This scale was compiled by Connor and Davidson (22) and mainly reflects the adaptive ability of individuals facing stress. The scale contains 25 questions, including tenacity, strength, and three dimensions of optimism. Items are rated on a 5-point Likert scale (0-4), with higher total scores indicating greater resilience. The scale demonstrates good psychometric properties, and in this study, its Cronbach’s α was 0.91.

#### Perceived social support scale

The scale was compiled by Zimet et al. (23). It contains 12 items and three subscales, including family support, friend support, and other support, to measure the degree of individuals’ perception of support from family, friends, and other people. A Likert 7-point scoring method is adopted. The higher the score, the higher the level of social support perceived by individuals. Cronbach’s α coefficient for this scale was 0.93.

#### Patient health questionnaire-9

Depressive symptom severity over the past two weeks was assessed using the 9-item Patient Health Questionnaire (PHQ-9), originally developed by Kroenke et al. (24). The Chinese version of the scale, which has been validated in the general population (25), was administered in this study. Each item is rated on a 4-point scale ranging from 0 (“Not at all”) to 3 (“Nearly every day”). Total scores range from 0 to 27, with established clinical cut-off points for interpretation: 0-4 (minimal), 5-9 (mild), 10-14 (moderate), 15-19 (moderately severe), and 20-27 (severe). The PHQ-9 demonstrated high internal consistency in this sample, with a Cronbach’s α of 0.87.

### Data collection

This study employed a cross-sectional survey design using self-administered questionnaires. Following the principle of informed consent, the researcher sent questionnaires to the participants on the spot, and the questionnaires used unified guidance to explain the research purpose and answer relevant requirements. The participants filled out the questionnaires independently and anonymously, which took approximately 15–20 min. Following data collection, all questionnaires underwent systematic quality screening by two psychological professionals. The verification process focused on identifying incomplete submissions (defined as those with >5% missing data) and invalid response patterns, including straight-lining, obvious regularity, or random responding. Questionnaires exhibiting any of these characteristics were classified as invalid and excluded from subsequent analysis. Of the 402 questionnaires distributed, 390 were determined to be valid, yielding a final response rate of 97.01%.

### Statistical analysis

Statistical analyses were performed using IBM SPSS Statistics (Version 28.0; IBM Corp.) and IBM SPSS Amos (Version 17.0; IBM Corp.). Descriptive statistics, Chi-square/Fisher’s exact tests (for categorical variables), and Pearson correlation were performed. Hierarchical linear regression analyses were conducted to explore predictive factors for adjustment disorder. Bootstrapping with 5000 samples was adopted to test mediation effects. Normality and other key statistical assumptions were verified and met. Significance was set at p < 0.05 (two-tailed).

### Common method deviation test

Harman’s single-factor test was used to analyze all questionnaire items. The results showed that there were 4 factors with eigenvalues greater than 1, and the variance explained by the first factor was 24.41%, which was less than the critical standard of 40%, indicating that no serious common method bias in this study.

## Results

### Occurrence of adjustment disorder among freshmen

**Table 1** presents the descriptive statistics and results of the univariate analyses. The results showed that adjustment disorder had no statistical significance according to participants’ gender, age, only child status, childhood trauma experience, and educational level (P>0.05). More than 50% of participants with adjustment disorder were from country (57.25%), and there was a significant association between adjustment disorder and place of growing up (P = 0.002), with a small effect size (Cramer’s V = 0.157, 95% CI [0.058, 0.257]). There was a significant correlation between adjustment disorder and family yearly income (P = 0.002; Cramer’s V = 0.167, 95% CI [0.067, 0.266]), including 87 cases (63.04%) from families with less than 100, 000 yuan and 51 cases (36.96%) from families with more than 100, 000 yuan. The association between childhood primary caregivers and adjustment disorder was significant (P = 0.008; Cramer’s V = 0.157, 95% CI [0.058, 0.256]); *post-hoc* comparisons indicated a higher incidence in the grandparents versus parents group (χ² = 6.46, p = 0.011).

**Table 1:** Adjustment disorder according to demographic characteristics (n=390).

Variable	No adjustment disorder	Adjustment disorder	χ^2^	P‐value
Overall	252	138		
Gender			0.112	0.738
Men	173	97		
Women	79	41		
Living situation			9.601	0.002
City	149	59		
Country	103	79		
Childhood primary caregivers			9.652	0.008
Parents	218	102		
Father or mother alone	15	15		
Grandparents or maternal grandparents	19	21		
Educational level			1.034	0.309
Professional high school	12	10		
Bachelor or college degree	240	128		
Family yearly income			10.815	0.001
Less than 100, 000 yuan	115	87		
More than 100, 000 yuan	137	51		
Childhood trauma experience			2.397	0.122
Yes	8	9		
No	244	129		
Only child in family			21.662	<0.001
Yes	123	101		
No	129	37		

### Correlation analysis

**Figure 1:**
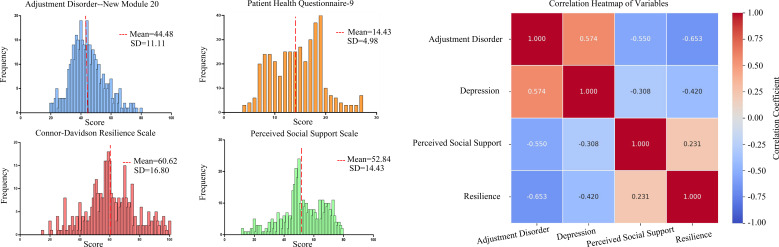
Histograms of scale scores and correlation matrix heatmap. SD, standard deviation. Histograms display score distributions for four measures: Adjustment Disorder-New Module 20, Patient Health Questionnaire-9, Connor-Davidson Resilience Scale, and Perceived Social Support Scale (mean and SD values are labeled for each). The heatmap presents pairwise correlation coefficients between variables (color intensity indicates correlation strength, with the legend representing correlation values).

The participants’ mean scores were 44.48 ± 11.11 for the ADNM-20, 60.62 ± 16.80 for the CD-RISC, 52.84 ± 14.43 for the PSSS, and 14.43 ± 4.98 for the PHQ-9. Pearson correlation analysis showed that perceived social support (r = -0.550, 95% CI [-0.620, -0.481], P <.001) and resilience (r = -0.653, 95% CI [-0.710, -0.596], P <.001) were significantly negatively correlated with adjustment disorder. Depression was positively correlated with adjustment disorder (r = 0.574, 95% CI [0.507, 0.641], P <.001), but negatively correlated with perceived social support (r = -0.308, 95% CI [-0.398, -0.218], P <.001) and resilience (r = -0.420, 95% CI [-0.502, -0.338], P <.001) (**Figure 1**).

### Regression analysis

We conducted a hierarchical linear regression analysis to examine the predictive effects of depression, resilience, and perceived social support on adjustment disorder, after controlling for demographic variables. The results are presented in **Table 2**. Model 1, which included demographic variables (Living situation, family yearly income, and childhood primary caregivers), was statistically significant and explained 6.5% of the variance in adjustment disorder (R² = 0.065, 95% CI for R² [0.025, 0.105]). Within this model, family yearly income (β = -0.153, 95% CI [-0.278, -0.028], P < 0.01) and childhood primary caregivers (β = 0.132, 95% CI [0.012, 0.252], P < 0.05) were significant predictors of higher levels of adjustment disorder.

**Table 2:** Hierarchical regression analysis of the relationships between depression, resilience, perceived social support, and adjustment disorder (n = 390).

Variable	Model 1					Model 2					Model 3					Model 4				
	B	SE	β	T	P	B	SE	β	T	P	B	SE	β	T	P	B	SE	β	T	P
Living situation	1.633	1.384	0.065	1.180	0.239	1.391	1.151	0.055	1.208	0.228	0.215	0.975	0.009	0.221	0.825	0.256	0.850	0.010	0.301	0.764
Family yearly income	-3.666	1.303	-0.153	-2.814	0.005	-2.601	1.087	-0.108	-2.394	0.017	-1.806	0.918	-0.075	-1.967	0.050	-0.827	0.805	-0.034	-1.027	0.305
Childhood primary caregivers	1.625	0.624	0.132	2.606	0.010	0.621	0.524	0.051	1.184	0.237	0.313	0.443	0.026	0.707	0.480	0.151	0.386	0.012	0.391	0.696
Depression						1.324	0.101	0.548	13.119	<0.001	0.861	0.093	0.357	9.305	<0.001	0.651	0.083	0.269	7.852	<0.001
Resilience											-0.338	0.027	-0.485	-12.563	<0.001	-0.312	0.024	-0.448	-13.241	<0.001
Perceived social support																-0.273	0.025	-0.355	-11.064	<0.001
R2			0.065					0.354					0.542					0.653		
△R2								0.289					0.188					0.111		
F	F(3, 387)=8.875					F(4, 386)=52.852					F(5, 385)=91.067					F(6, 384)=120.282				

B, Unstandardized regression weight; SE, Standard error for B; β, Standardized beta weight; P, statistical significance.

The subsequent addition of depression, resilience, and finally perceived social support significantly improved the model. In the final model 4, depression (β =0.269, 95% CI [0.139, 0.399], P <0.001), resilience (β = -0.448, 95% CI [-0.524, -0.372], P <0.001), and perceived social support (β = -0.355, 95% CI [-0.431, -0.279], P <0.001) all showed significant independent contributions to adjustment disorder.

### Structural model analyses

To explore the specific mediating roles of resilience and perceived social support in the relationship between depression and adjustment disorder, a parallel mediation model was constructed. Depression was specified as the independent variable, adjustment disorder as the dependent variable, and resilience and perceived social support as parallel mediating variables. The model’s goodness-of-fit indices indicated an acceptable fit to the data (see **Table 3** for details).

**Table 3:** Fit index of the mediation model.

Index	χ^2^	df	χ^2^/df	GFI	AGFI	CFI	TLI	NFI	RMSEA
Value	4.339	1	4.339	0.993	0.931	0.976	0.949	0.972	0.096

GFI, goodness-of-fit index; AGFI, adjusted goodness-of-fit index; CFI, comparative fit index; TLI, Tucker–Lewis index; NFI, normed fit index; RMSEA, root mean square error of approximation.

**Figure 2:**
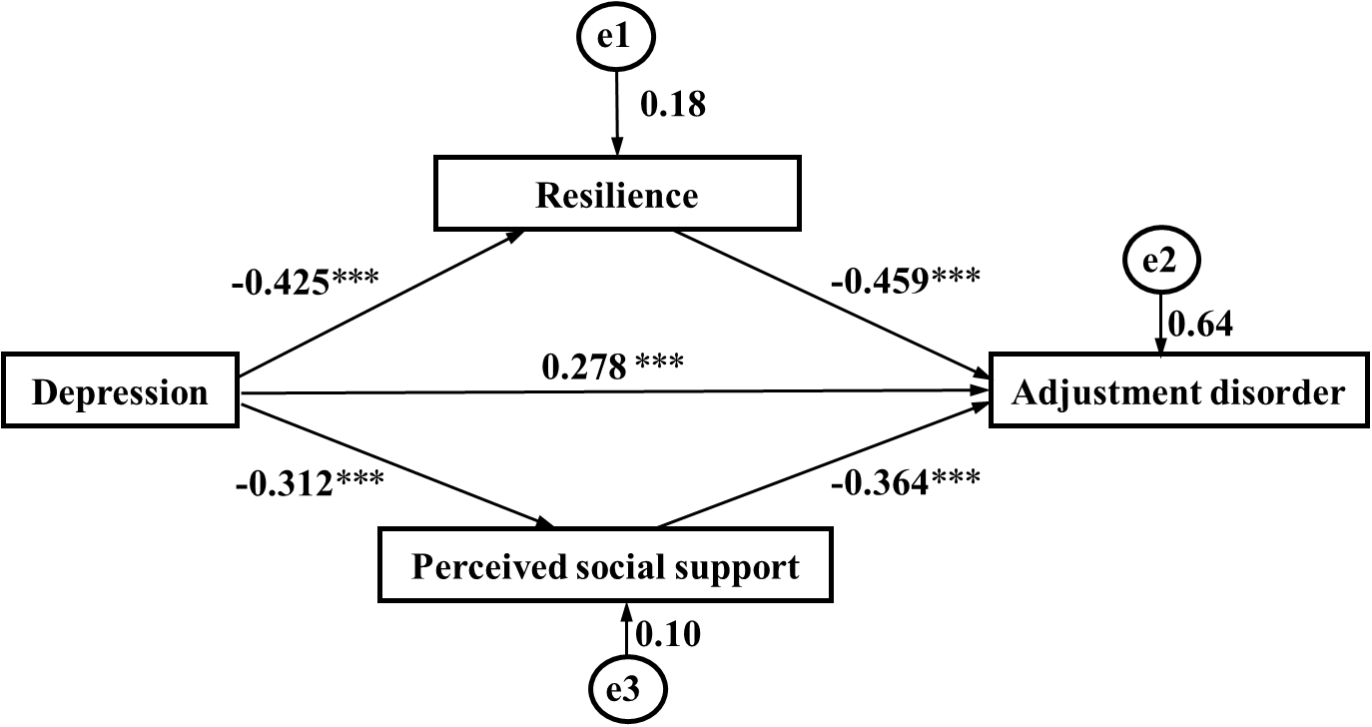
Path diagram of the parallel mediation model. Note: All coefficients are standardized and statistically significant at the 0.05 level (***p < 0.001).

The path coefficients, along with their 95% confidence intervals derived from 5000 bootstrap samples, are presented in **Table 4** and **Figure 2**. All hypothesized paths were statistically significant, as indicated by 95% CIs excluding zero. Specifically, depression showed a significant positive association with adjustment disorder (β = 0.278, 95% CI [0.213, 0.342], P < 0.001) and a negative association with both resilience (β = -0.425, 95% CI [-0.504, -0.327], P < 0.001) and perceived social support (β = -0.312, 95% CI [-0.420, -0.197], P < 0.001). In turn, both resilience (β = -0.459, 95% CI [-0.536, -0.376], P < 0.001) and perceived social support (β = -0.364, 95% CI [-0.436, -0.294], P < 0.001) were each significantly and negatively associated with adjustment disorder. These results confirm the significant mediating roles of resilience and social support.

**Table 4:** Bootstrap analysis of direct, indirect, and total effects (n=390).

Path	Estimate	Boot SE	Bias-Corrected 95% CI		Effective size/%
			Lower	Upper	
Direct effect: depression → adjustment disorder	0.656	0.086	0.503	0.836	47.3
Indirect effect1: depression→ resilience→adjustment disorder	0.461	0.061	0.353	0.596	33.3
Indirect effect 2:depression→perceived social support → adjustment disorder	0.269	0.051	0.173	0.371	19.4
Total effect	1.386	0.109	1.171	1.600	100

Bootstrap sample size = 5000; SE = standard error; CI = confidence interval. All estimates are standardized path coefficients. All 95% bias-corrected confidence intervals do not contain 0, indicating statistically significant effects (p < 0.05).

Based on the above, bootstrapping techniques were used to evaluate multiple mediation. The original data (n=390) were randomly sampled 5000 times, and the Bootstrap 95% confidence interval of the mediating effect was calculated. As seen in **Table 4**, the effect values of the two intermediary paths were both within the confidence interval, and the upper and lower limits of the confidence interval did not contain 0; therefore, the intermediary effect was significant. The results showed that the indirect effect of depression on adjustment disorder was 0.730 (0.461 + 0.269), and the effect size was 52.7%. The direct effect was 0.656 and the effect size was 47.3%. The results showed that depression not only predicts adjustment disorder directly, but can also predict it through the mediating effect of resilience and perceived social support.

## Discussion

The results of this study showed that freshmen from the country, those raised by older generations, and those from families with poor economic status had higher levels of adjustment disorder, which is consistent with Sheldon and Ababu’s findings (5, 26). Attachment theory (27) holds that, in the early years of a person’s life, if the main caregiver is unable to provide a sustainable and stable upbringing owing to reasons such as work or divorce, it may cause mental damage to the child. These children may lack frustration coping ability, face difficulties in adapting to society, and be more prone to interpersonal difficulties, emotional difficulties, and other adaptation problems (10). Our study found that adjustment disorder had no statistical significance in terms of gender, age, only child status, childhood trauma experience, and educational level. Previous studies have found that being female, age, and being an only child are risk factors for adjustment disorder (28). There is also a large amount of evidence that women and only children have a higher risk of mental disorders (29). Therefore, whether there are differences in the adaptability of college students in gender, age, place of origin, and family structure is worthy of further study, which may be related to differences in sampling methods, study groups, and regions. There was a significant correlation between adjustment disorder and the place of growing up, primary caregivers in childhood, and family economic situation, suggesting that we should pay attention to the family economic situation of college students who grew up in rural areas and were raised by grandparents.

This study focused on the relationship between depression, resilience, perceived social support, and adjustment disorder among college freshmen, and found that adjustment disorder was positively correlated with depression, which is consistent with previous research (26). Developmental psychology postulates that negative emotions and adjustment problems accompany each stage of life development, and the university stage may be a more prominent period for adjustment problems. College freshmen are directly faced with academic pressure, environmental changes, social adaptation, role transformation, and other burdens, and have poor self-adjustment and recovery abilities. They are unable to adapt to the pressure and thus experience anxiety, depression, and other negative emotions, accompanied by changes or disorders in cognition, behavior, psychophysiology, and interpersonal relationships, resulting in plummeting levels of resilience and emergence of adjustment disorder (3, 30).

In addition, studies have shown that resilience and perceived social support in college freshmen are negatively correlated with depression and adjustment disorder. Based on the stress-cognitive susceptibility model, the interaction between environmental stress factors and individuals’ intrinsic characteristics predicts the onset and severity of depression. Environmental factors include stress during college, negative life events, environmental adaptation, and social support. Intrinsic qualities include resilience, personality factors, and coping styles (10). Therefore, resilience and perceived social support serve as important mediating factors in the relationship between perceived stress and adjustment disorder. The trait theory of resilience postulates that resilience is a personality trait or capacity enabling individuals to respond effectively to negative life events, such as stress, frustration, and trauma, and is a key protective factor for mental health. One study (30) reported that resilience scores were significantly negatively correlated with the severity of depressive symptoms in patients with depression. Other studies (31) have shown that individuals can use resilience to reduce psychological stress and associated negative emotions, thereby maintaining and improving their quality of life.

Similar to other groups, college freshmen experience depression and other negative emotions after facing stressful life events. The more social support they receive or perceive, the stronger their adaptive ability will be, which can prevent and alleviate adjustment disorder caused by the stress response. Studies have found that social support is associated with positive health effects (32), and that support and assistance from the family, community, and government are critical to reducing the negative impact of disasters on individuals’ mental health (33). The results of the regression analysis in this study showed that depression, resilience, and perceived social support were factors influencing adjustment disorder. Therefore, to study the resilience of patients with adjustment disorder from the perspective of positive psychology and mobilize the inherent potential of resilience and social support has practical significance for reducing the risk of adjustment disorder onset and promoting the application of positive intervention measures.

Interestingly, this study found that resilience and perceived social support played a partial mediating role in the relationship between depression and adjustment disorder among college freshmen. The results of the path analysis showed that resilience had a significant indirect effect on the relationship between depression and adjustment disorder, with an standardized indirect effect of 0.461, accounting for 33.3% of the total effect. Similar to the findings of Windle (31), resilience, as a patient’s ability to recover quickly after experiencing negative events, can effectively reduce individual stress levels and the negative impact of stressful events. When encountering stressful life events, individuals with high resilience can adopt positive coping styles, make accurate and positive cognitive evaluations and behavioral adjustments, deal appropriately with pressure and difficulties, and transform pressure into motivation (34). In addition, individuals with high resilience can quickly adapt to the changing environment, recover from pressure, and reduce adjustment difficulties. The inverse relationship between resilience and symptoms of depression and anxiety following adversity has been well established (30), and the effectiveness of resilience-based interventions in mitigating negative emotions has also been validated (35). During university, students face various pressures, including academic demands, negative life events, interpersonal issues, and challenges related to role transition, self-orientation, and personal development. As a protective capacity for coping with stressful situations, resilience can help college students enhance positive psychological attributes and alleviate adjustment disorder symptoms resulting from depression.

In the present study, social support also served as a mediator between depression and adjustment disorder. According to self-determination theory, which integrates basic psychological needs theory, both the individual and the environment play crucial roles in development. When the environment satisfies individuals’ basic psychological needs, their well-being and personal growth are enhanced. Numerous studies have shown that perceived social support plays a protective role in mental health (36, 37). The higher the level of resilience in patients with depression, the higher the perception of social support, which is conducive to living a positive life and facing difficulties, and ultimately reduces the possibility of adjustment disorders. Previous research has also revealed the role of social support as a resilient factor (19, 37). In theory, individuals who utilize social support can adapt to or change external stressors, thereby facilitating their adjustment and achieving better psychosocial functioning.

Based on a clinical sample of college freshmen, this study not only confirmed that resilience and perceived social support play important mediating roles in the relationship between depression and adjustment disorder but also revealed the buffering mechanism against depression through positive psychological resources (i.e., resilience) and perceived social support. Enhancing resilience and perceived social support in this clinical population is of great significance for alleviating adjustment disorder symptoms. Therefore, interventions targeting college freshmen with depression should aim to foster resilience and strengthen perceived social support. Developing a multidimensional support system that helps students mobilize social support networks and adopt positive coping strategies may improve their health and quality of life. Personalized interventions that enhance individuals’ ability to perceive and utilize social support are recommended to collectively reduce adjustment difficulties.

There are several important limitations in this study that should be acknowledged. First, the cross-sectional design prevents us from establishing causal relationships between the studied variables. Future research would benefit from longitudinal approaches to better understand the temporal dynamics and causal pathways in adjustment disorder development. Second, our reliance on self-reported measures without supplementary verification through clinical interviews may affect the accuracy of mental health assessments. The rates of psychological outcomes reported here should therefore be interpreted with caution. Third, and most importantly, the generalizability of our findings is substantially limited by our sampling method. Participants were recruited through a hospital’s Pediatric Psychology Department, resulting in a clinical sample that demonstrated a high prevalence of adjustment disorder (35.4%). This sample characteristic means our results are not representative of the general college freshman population and should not be generalized beyond similar clinical contexts. Future studies should consider incorporating additional relevant factors and exploring different modeling approaches to better capture the complexity of adjustment disorder.

## Conclusions

This study, conducted within a clinical sample of college freshmen seeking psychological services, demonstrates that resilience and perceived social support significantly mediate the relationship between depression and adjustment disorder. Our findings indicate that depression affects adjustment disorder both directly and indirectly by undermining resilience and the perception of social support. These results underscore the potential of interventions aimed at bolstering these resources to alleviate psychological distress in this clinical population. Future studies should utilize longitudinal designs to confirm causality and explore these mechanisms in broader, non-clinical samples, while considering moderators such as gender and socioeconomic status.

## Data Availability

The original contributions presented in the study are included in the article/supplementary material. Further inquiries can be directed to the corresponding author.
